# Bioaerated Low-Density Composites from Industrial Byproducts: Advancing Carbon-Neutral and Energy-Efficient Material Systems in the Building Sector

**DOI:** 10.3390/ma19132722

**Published:** 2026-06-25

**Authors:** Corradino Sposato, Tiziana Cardinale, Andrea Feo, Francesco Catucci, Maria Bruna Alba

**Affiliations:** Energy Technologies and Renewable Sources Department (TERIN), Italian National Agency for New Technologies, Energy and Sustainable Economic Development (ENEA), Trisaia Research Center, S.S. 106 Ionica, km 419+500, 75026 Rotondella, Italy; corradino.sposato@enea.it (C.S.); andrea.feo@enea.it (A.F.); francesco.catucci@enea.it (F.C.); mariabruna.alba@enea.it (M.B.A.)

**Keywords:** energy efficiency, cementitious materials, aerated concrete, lightweight cellular concrete, foaming agent, air void structure, durability, mechanical strength, thermal insulation, sustainability

## Abstract

The transition towards carbon-neutral construction materials requires innovative solutions that combine reduced embodied energy, enhanced durability and improved building energy efficiency. This study investigates and compares two novel bioaerated low-density composites—BAAC and BIOAERMAC—developed through biologically driven aeration processes incorporating industrial byproducts. BAAC is produced using Saccharomyces cerevisiae and hydrogen peroxide, replacing conventional aluminum powder and improving safety while enabling the valorization of waste-derived yeast. BIOAERMAC is a gypsum-based composite incorporating synthetic anhydrite, microorganisms, peroxides, and recycled rubber from end-of-life tires. The materials were characterized in terms of hygrothermal behavior and dimensional stability, and compared with commercial autoclaved aerated concrete under equivalent mechanical strength conditions. The results highlight significant differences in moisture transport and shrinkage, primarily governed by pore structure and connectivity. BAAC exhibits behavior comparable to conventional AAC, whereas BIOAERMAC shows reduced capillary and hygroscopic absorption, indicating limited pore connectivity, but higher drying shrinkage. These findings demonstrate the effectiveness of bioaeration in tailoring pore structure and controlling the trade-off between moisture transport, durability, and dimensional stability, highlighting the potential of bioaerated composites for low-carbon and energy-efficient building applications.

## 1. Introduction

The construction sector today faces a dual challenge: on the one hand, reducing the climate footprint associated with materials, particularly embodied carbon and embodied energy [1,2,3,4]; on the other, increasing energy efficiency and durability of the built environment throughout its entire life cycle. Numerous studies highlight that simply reducing energy consumption during the use phase is no longer sufficient and that true decarbonization requires an integrated life cycle approach, capable of jointly considering materials, construction processes, operational performance, and the end-of-life of the building [1,5,6]. At the European level, this trajectory is explicitly framed by the New Circular Economy Action Plan (COM (2020)98), which dedicates a specific focus to “Construction and buildings” and strengthens the orientation toward durability, adaptability, reuse, and the valorization of secondary resources [7]. In parallel, methodological frameworks and synthesis studies on embodied carbon highlight that the decarbonization of buildings cannot be based solely on operational energy consumption but must include material selection and process strategies that act effectively both “upstream” (extraction, production and logistics) and “downstream” (end-of-life and reintegration into circular cycles) [8,9].

In this context, lightweight and porous materials play a strategic role because they reduce density and thermal conductivity, enhancing the thermal insulation performance of the building envelope and potentially benefiting it in terms of permanent loads and construction uses. For systems based on autoclaved aerated concrete (AAC), the most recent literature shows how mechanical and thermal properties are strongly governed by density, porosity, and microstructure, and how overall sustainability depends jointly on on-site performance and characteristics of the composition and production process [10,11,12,13,14]. Consistently, reviews dedicated to lightweight materials obtained from agro-industrial and industrial byproducts emphasize that the use of secondary resources can reduce the use of raw materials but also introduces new challenges in terms of porosity control, water absorption, and dimensional stability [15,16,17,18,19]. Consequently, the design of low-emission cellular materials requires a rigorous balance between thermal performance, mechanical requirements, and microstructural quality.

Porosity control is, in fact, a key issue for cellular materials. Studies focused on the characterization of pore structure in cellular materials show that porosity, void size and distribution, as well as connectivity, directly influence mechanical behavior and heat and moisture transport [20,21,22,23,24]. In this sense, the management of the kinetics of gas bubble formation and stabilization represents a crucial point: in autoclaved aerated concrete and cellular concrete, the “quality” of aeration, understood as the graduality of gas release, stability of the system in the fresh phase and the ability to preserve homogeneous porosity during setting, is crucial to achieve an adequate balance between density, hygroscopic absorption and resistance [12,21,24,25,26,27].

The present work aims to correlate pore structure with hygrothermal transport, energy efficiency, and dimensional stability, highlighting the role of microstructure in improving the overall performance of low-density building materials under equivalent mechanical conditions.

## 2. Materials and Methods

This section presents the two innovative materials, BAAC and BIOAERMAC, along with a definition of the critical issues related to their thermohygrometric behavior and a description of the experimental program.

### 2.1. Bioaeration for Porosity Control in Cellular Materials

Within this framework, this paper proposes and compares two innovative materials developed in ENEA laboratories, and subsequently patented, namely Bio Aerated Autoclaved Concrete (BAAC) [28] and BIOAERMAC (BIO-AERation of Calcium Sulfate-Based Composite Materials) [29]. These materials share a distinctive feature: a biologically assisted aeration mechanism (“bioaeration”) designed to generate a porous structure, reducing dependence on conventional aerating agents typical of industrial processes.

BAAC [30] was born as a sustainable evolution of AAC and addresses a widely recognized criticality: the use of aluminum powder as an aerating agent, associated with safety, process management, and environmental impact issues. In BAAC, aeration is achieved through the dismutation reaction of hydrogen peroxide catalyzed by biological systems associated with Saccharomyces cerevisiae, with the release of oxygen in the form of bubbles and consequent volumetric expansion and formation of porosity. The material was produced in density classes ranging from approximately 350 to 850 kg/m^3^ and characterized physically, thermally, and mechanically, displaying compressive strengths ranging from 1 to 9 MPa and thermal conductivities ranging from just under 0.10 W/mK to 0.30 W/mK.

BIOAERMAC [31] extends the same bioaeration technological paradigm to a gypsum matrix based on synthetic anhydrite—a byproduct of hydrofluoric acid production—combined with sulfoaluminate cements and, in some formulations, with rubber granules from end-of-life tires (ELT). Here too, the fresh gas is generated by a system composed of microorganisms and peroxides that produces trapped oxygen during setting, allowing for controlled lightweighting. The resulting densities range between 600 and 950 kg/m^3^, with compressive strengths of up to approximately 6 MPa and thermal conductivities in the range of 0.15–0.30 W/mK, highlighting the composite’s competitiveness compared to commercial lightweight products. Furthermore, the results show that the introduction of ELT can improve thermal performance at the same density, despite a decrease in strength attributed to the weakness of the rubber-matrix interface. This behavior is consistent with literature research on the use of recycled rubber in lightweight and sustainable concrete, where the improvement in some functional performances (including thermal or dynamic response) is often accompanied by a reduction in strength due to the nature of the interface and lower adhesion [32,33,34].

**Figure 1:**
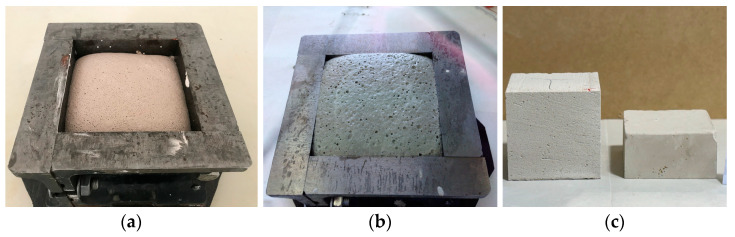
(**a**) BIOAERMAC; (**b**) BAAC; (**c**) comparison of aerated and not aerated product.

The comparison between BAAC and BIOAERMAC (Figure 1) is therefore scientifically relevant because it brings together two families of materials—the first based on an autoclaved cementitious matrix and the other on a non-autoclaved gypsum/sulfoaluminate matrix—which converge on the same technological solution: bioaeration as a tool for generating and controlling porosity. In the literature on autoclaved aerated concrete, and on cellular concretes more generally, there are numerous attempts to replace or integrate traditional air-entraining agents with alternatives derived from industrial byproducts; in these approaches, the control of gas evolution kinetics is recognized as a key factor for stabilizing volume, density and mechanical properties [35]. Similarly, the use of metallurgical byproducts (e.g., salt slag) as gas sources/activators in alkaline-activated cellular concretes confirms that the generation of “internal” porosity is an effective lever for combining lightweighting and thermal insulation but requires careful microstructural management to avoid collapse and performance losses [36]. From this perspective, the bioaeration proposed in BAAC and BIOAERMAC [28,29,30,31] appears particularly interesting because it aims to achieve more controllable and potentially safer bubble production compared to systems based on metal reagents and/or foams, while maintaining the performance capabilities of cellular materials [35,37,38,39]. From a mechanical and thermal perspective, both materials exhibit the typical performance of cellular materials: as density increases, mechanical resistance tends to increase, and, concurrently, thermal conductivity increases due to the reduction in the trapped air fraction.

On this basis, a comparative framework between BAAC and BIOAERMAC can be established, whose main characteristics are summarized in the following Table 1:

This interpretative framework is consistent with the behavior of AAC, whose macroscopic properties are linked to the porous microstructure and density–porosity relationships. Controlling porosity is crucial to achieving an optimal combination of strength and insulation performance and, on the other hand, the need to integrate environmental and mechanical parameters into joint indicators (eco-mechanical) for a more comprehensive sustainability assessment of AAC blocks [40,41].

The innovation featured in these two solutions not only offers two innovative materials but also two technological systems that enable strategies consistent with circularity and decarbonization, in line with European frameworks and the principles of embodied carbon reduction [7,40,42].

The paper, starting from a comprehensive comparison between BAAC and BIOAERMAC from a mechanical and thermal point of view, then extends the analysis to parameters that are crucial for the durability and real-life use of porous materials, focusing on hygroscopic absorption and dimensional stability (shrinkage), aspects that the scientific literature identifies as strongly dependent on porosity and its morphology [8,26].

### 2.2. Microstructural Characterization

Microstructural characterization was performed using an optical microscope (Leica Microsystems DVM6, Leica, Wetzlar, Germany) equipped with a medium magnification lens and coaxial illumination, with a maximum field of view of 12.55 mm. Image acquisition and processing were carried out using Leica Application Suite X software, version 5.2.0.26130, released for 2022 (Leica, Wetzlar, Germany).

Optical microscopy combined with image analysis was used to investigate the pore system of the hardened material, with particular attention to pore size distribution, morphology, and spatial arrangement.

Images were acquired at a magnification of 10×, selected as a compromise between resolution and representativeness of the pore system.

### 2.3. Hygrothermal Performance and Dimensional Stability

However, the optimization of cellular materials cannot be based exclusively on density, strength, and thermal conductivity: the porosity that ensures insulation and lightness also governs the moisture transport mechanisms and the material’s hygroscopic response. Indeed, the literature highlights how the porous structure (pore size/distribution and connectivity) directly affects not only mechanical and thermal properties but also water absorption and migration phenomena in cellular composites [20]. At the same time, numerous reviews on materials lightweighted with industrial byproducts highlight the need to carefully evaluate water absorption and dimensional stability, since the introduction of new phases and microstructural configurations can alter porosity and permeability, modifying the material’s sensitivity to humidity [8]. This issue is particularly relevant in porous cementitious composites, where the correlations between density, porosity, and macroscopic behavior are central, and the durability of the building system depends largely on moisture transport phenomena and the material’s response to real-world temperature and humidity conditions [23,43,44,45,46].

In the case of the two composites analyzed here, these aspects are even more pertinent, as the innovation lies precisely in the porosity generation mechanism and therefore requires specific investigations into hygrothermal performance and long-term response, explicitly including dimensional stability and shrinkage as future development directions. In this sense, recent evidence on cellular materials and foamed concretes shows how variations in the microstructure and porosity system are directly reflected in measurable changes in drying shrinkage, as well as in thermal properties, highlighting the role of pore distribution, moisture transport phenomena, and environmental conditions in controlling deformation and material durability [42,47,48,49,50,51].

Considering this, in the following sections, the BAAC–BIOAERMAC comparison is extended to include hygroscopic absorption and shrinkage. The aim is to clarify how different binding matrices, and especially how specific bioaeration modes and resulting pore morphology, influence the balance between thermomechanical performance and dimensional-hygroscopic response, with a view to fully reliable application and industrial transferability.

### 2.4. Experimental Program

This study analyzed three material systems: Bio Aerated Autoclaved Concrete (BAAC), BIOAERMAC composite based on gypsum–CSA system, and, for comparative purposes, a selected block of commercial autoclaved aerated concrete (GASBETON^®^ Evolution, EKORU s.r.l., Volla (NA) Italy [52]) and reported below as commercial AAC, widely used in the construction industry and representative of the solutions available on the market.

The comparison was conducted on materials with comparable mechanical strength (approximately 3 MPa). This choice is based on three main considerations.

On the one hand, a performance-based approach was adopted, favoring the comparison of materials intended for the same construction use rather than materials with similar physical properties but different applications. Mechanical strength is, in fact, the most direct reference parameter for identifying the intended use. Indeed, materials with compressive strengths in the range of 2–4 MPa are typically used in non-load-bearing elements, such as lightweight infill walls and internal partitions.

On the other hand, comparing materials with equal strength allows us to more clearly highlight the role of the microstructure and the mode of formation of porosity, which is the distinguishing feature of the materials studied. Given equal mechanical performance, any differences in terms of absorption and shrinkage can be more reliably attributed to the distribution and connectivity of the pores.

Finally, the choice is justified by its application relevance: the materials considered are intended for non-load-bearing building components, for which hygrothermal and dimensional performance play a crucial role: water absorption can affect insulation and durability, while shrinkage is often responsible for cracking and incompatibility with finishing systems. Consequently, comparing materials with equal mechanical performance levels is not only methodologically correct but also engineeringly significant.

BAAC specimens with a density of approximately 500 kg/m^3^ and mechanical strength of around 3 MPa were therefore selected, along with BIOAERMAC specimens with a density class of approximately 800 kg/m^3^ and mechanical strength of around 3 MPa. For commercial AAC, the choice fell on blocks belonging to typical density classes of approximately 500 kg/m^3^, with thermal conductivity λ equal to 0.110 W/mK and a compressive strength value of 3.5 MPa [52]. These materials are designed for applications in non-load-bearing elements, such as partitions, internal partitions, and lightweight infill panels, and are characterized by a highly porous cellular structure developed through controlled industrial processes. From a hygrothermal perspective, the materials considered exhibit:

- a vapor resistance factor μ typically between 5 and 10;
- high vapor permeability;
- marked sensitivity to environmental conditions, requiring, as indicated in the technical data sheets, adequate protection during installation to prevent imbibition or premature degradation.

Furthermore, the volumetric deformation values associated with humidity variations are in the order of ±0.2–0.4 mm/m, confirming the relevance of hygrometric shrinkage for this class of porous materials.

The use of these materials as an experimental reference provides a consolidated benchmark, characterized by well-known and standardized properties, against which to evaluate the performance of the innovative BAAC and BIOAERMAC composites. In particular, the comparison highlights the role of different porosity generation methods on moisture transport and dimensional stability, while maintaining a direct link with real-world applications in the construction sector.

It is also important to emphasize that BAAC and BIOAERMAC represent innovative materials, not only not yet available on a commercial scale, but also not fully codified within specific technical standards. For this reason, it was deemed necessary to compare them with well-established reference materials, such as commercial AAC, using recognized testing standards that were as consistent as possible with the nature of the materials analyzed. In this sense, the regulatory choice was guided by the need to ensure both the comparability of the results and their practical relevance.

#### 2.4.1. Hygroscopic Sorption Properties and Capillary Water Absorption

The characterization of moisture behavior was conducted through the combined application of the following standards:

- UNI EN ISO 12571:2022—Hygrothermal performance of building materials and products—Determination of hygroscopic sorption properties [53];
- UNI EN 772-11:2011—Test methods for masonry units—Determination of capillary water absorption and initial absorption rate [54].

The combined use of these standards was motivated by the need to comprehensively describe the hygrothermal behavior of porous materials, considering the different mechanisms of interaction with water.

The UNI EN ISO 12571:2022 standard allows for the evaluation of hygroscopic sorption, or the material’s ability to adsorb water vapor as a function of the relative humidity of the environment. This phenomenon is strongly influenced by microporosity and specific surface area and is representative of typical operating conditions in indoor environments. The test therefore provides fundamental information on the material’s behavior in hygrothermal equilibrium and on potential dimensional changes induced by humidity. At least three specimens were tested for each material; the selected sample size shall consider any heterogeneity of the materials.

In contrast, the UNI EN 772-11:2011 standard is aimed at determining water absorption in the liquid phase due to capillarity. This type of test is directly related to open porosity, pore size, and their connectivity, and simulates more severe conditions, such as exposure to driving rain or capillary rise. The results obtained are therefore particularly relevant for assessing the material’s durability.

According to the standard, six specimens were tested, corresponding to representative masonry units. The standard does not prescribe fixed specimen dimensions, as the test is conducted on elements with their original geometry to preserve the capillary absorption mechanism. The combined use of the two methodologies allows us to distinguish between:

- vapor-phase transport phenomena, associated with micropores (UNI EN ISO 12571:2022);
- liquid-phase absorption phenomena, linked to capillary and interconnected porosity (UNI EN 772-11:2011).

This distinction is crucial for cellular materials, where the response to humidity is strongly dependent on the pore size distribution and its interconnection. Using both tests, therefore, allows for a more complete and representative characterization of actual service behavior.

#### 2.4.2. Drying Shrinkage

The drying shrinkage assessment was performed according to UNI EN 680:2006—Determination of drying shrinkage of autoclaved aerated concrete [55]. The test set shall consist of three prisms with a cross-section of 40 mm × 40 mm and a length to suit the length of the measuring apparatus, but not less than 160 mm.

Although UNI EN 680:2006 was originally developed for autoclaved aerated concrete (AAC), its adoption in this study is justified by the similarity in the governing mechanisms of dimensional changes in highly porous, low-density materials. In both AAC and the investigated BIOAERMAC, drying shrinkage is primarily driven by moisture loss within a cellular pore network, leading to the development of capillary stresses and associated microstructural deformation. Therefore, despite differences in binder chemistry and curing process, the dominant shrinkage mechanisms remain comparable. On this basis, the standard was considered applicable as a physically consistent method for evaluating shrinkage response in materials characterized by high porosity, large specific surface area, and moisture-controlled dimensional response.

## 3. Results

### 3.1. Pore Structure Characterization and Microstructural Analysis

Microstructural characterization [56,57,58] was performed to analyze the pore system of the investigated materials, with the aim of supporting the interpretation of moisture transport and shrinkage.

**Figure 2:**
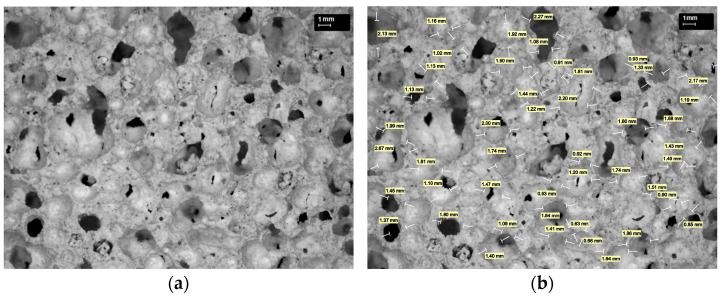
Microscope images of BAAC (10× magnification) without indication of pore diameter (**a**) and with indication of pore diameter (**b**).

**Figure 3:**
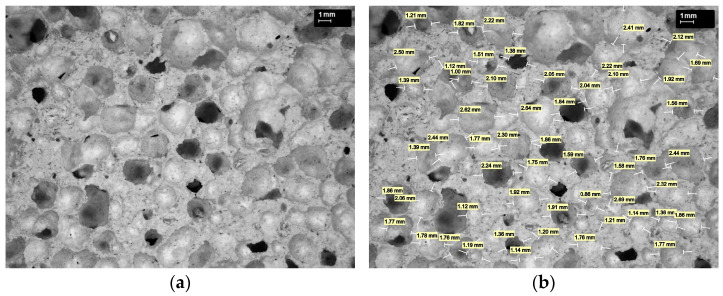
Microscope images of commercial AAC (10× magnification) without indication of pore diameter (**a**) and with indication of pore diameter (**b**).

**Figure 4:**
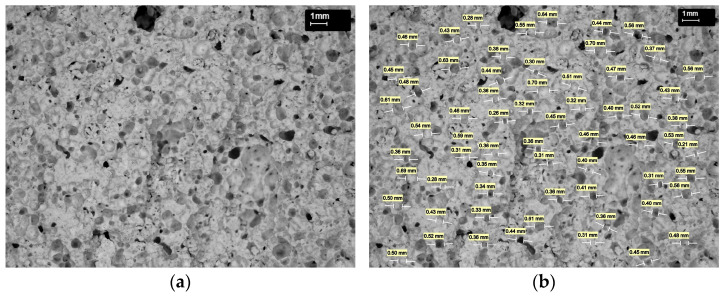
Microscope images of BIOAERMAC (10× magnification) without indication of pore diameter (**a**) and with indication of pore diameter (**b**).

The microstructural analysis shows a clear transition from a highly interconnected and heterogeneous pore network in AAC (average pore diameter =1.79 ± 0.46 mm) to a more uniform but still connected structure in BAAC (=1.53 ± 0.48 mm), and finally to a fine and weakly interconnected pore system in BIOAERMAC (=0.44 ± 0.11 mm) (Figure 2, Figure 3 and Figure 4).

Specifically, BIOAERMAC is characterized by smaller, more spherical, and more uniformly distributed pores, resulting in reduced pore connectivity and a more discontinuous transport network.

### 3.2. Hygrothermal Performance of Porous Materials

#### 3.2.1. Evaluation of Hygroscopic Sorption

**Figure 5:**
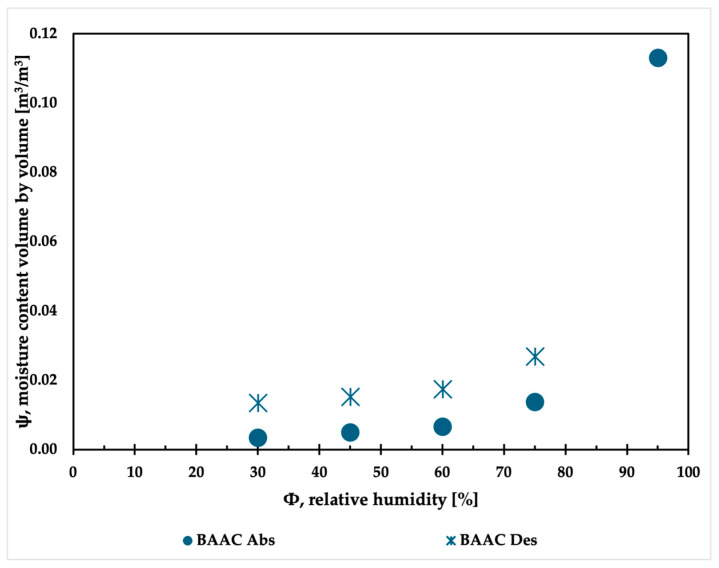
Equilibrium moisture content curve volume by volume for BAAC.

**Figure 6:**
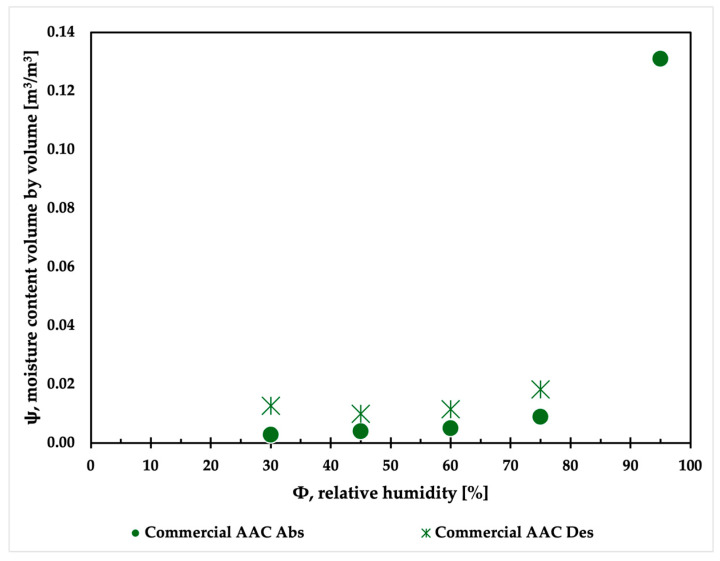
Equilibrium moisture content curve volume by volume for commercial AAC.

**Figure 7:**
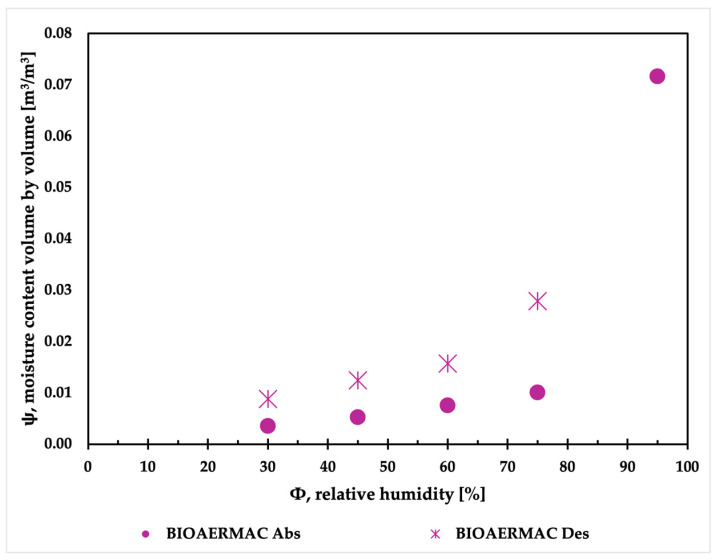
Equilibrium moisture content curve volume by volume for BIOAERMAC.

The sorption and desorption isotherms obtained according to UNI EN ISO 12571:2022 provide a comprehensive description of the hygroscopic performance of the investigated materials, enabling a detailed comparison between the innovative BAAC and BIOAERMAC and the commercial autoclaved aerated concrete, as reported in Table 2 and Table 3, Tables S1 and S2, and in Figure 5, Figure 6, Figure 7 and Figures S1–S3.

According to the standard methodology, these curves represent the equilibrium moisture content of porous materials under controlled relative humidity conditions, thus constituting a fundamental parameter for evaluating hygrothermal performance and durability in building applications. In the reported graphs, the X axis represents the relative humidity, while the Y axis shows the moisture content of the investigated materials expressed as a volumetric ratio (m^3^/m^3^). This representation was adopted because expressing moisture content on a mass basis, as commonly done and prescribed by the standard, would not be fully representative due to the different densities of the materials under investigation.

The adsorption curves exhibit an increase in volumetric moisture content with increasing relative humidity, which is consistent with the typical response of highly porous materials such as AAC, where moisture accumulation is governed by multilayer adsorption and capillary condensation within the pore network. However, significant differences emerge among the three materials, particularly at high relative humidity levels. Commercial AAC reaches the highest moisture content (approximately 0.13 m^3^/m^3^ at 95% RH), followed by BAAC (around 0.11–0.12 m^3^/m^3^), whereas BIOAERMAC shows a significantly lower moisture uptake (approximately 0.07 m^3^/m^3^). This trend can be directly attributed to the influence of bulk density on pore structure: lower-density AAC materials typically exhibit higher total porosity, resulting in a larger volume of accessible pores and consequently higher moisture storage capacity. Conversely, the higher density of BIOAERMAC implies a more compact microstructure with reduced pore volume, limiting both capillary condensation and vapor adsorption phenomena [59].

In the intermediate humidity range (30–75% RH), BAAC and commercial AAC display a more pronounced increase in moisture content compared to BIOAERMAC, indicating a higher hygroscopic sensitivity. This trend probably suggests that the pore size distribution of the low-density materials includes a greater fraction of pore interconnection, which are particularly active in the hygroscopic range and strongly influence sorption kinetics.

A clear hysteresis loop is observed between adsorption and desorption curves for all three materials. Specifically, the desorption curves are located above the adsorption ones over the entire range of relative humidity. This means that, for the same relative humidity value, the moisture content during desorption is higher than during adsorption. This behavior is due to the complex pore network, where some pores with narrow openings retain moisture and delay its release during drying [60].

Hysteresis is particularly pronounced in BAAC, moderately evident in commercial AAC, and less significant in BIOAERMAC. A larger hysteresis loop indicates a greater degree of water entrapment and a more heterogeneous pore structure characterized by interconnected pores.

From a material performance perspective, these results highlight that BAAC and commercial AAC, due to their higher moisture storage capacity and pronounced hysteresis, may provide enhanced moisture buffering capacity, which is beneficial for indoor humidity regulation and hygrothermal comfort. However, this also implies a potentially higher susceptibility to moisture accumulation under prolonged high-humidity conditions, with possible implications for durability and thermal performance, as moisture is known to significantly affect both properties in AAC-based materials. On the other hand, BIOAERMAC, characterized by lower moisture uptake and reduced hysteresis, appears to offer greater stability in humid environments; however, with a reduced capacity for passive humidity regulation.

#### 3.2.2. Determination of Water Absorption Due to Capillarity Action

The transport properties of porous materials play a key role in determining their durability. In the present study, capillary water absorption tests were carried out in accordance with UNI EN 772-11:2011.

Table 4 reports the results of the capillary absorption tests for the three investigated materials. In particular, *Cw,s* represents the coefficient of water absorption due to capillary action of autoclaved aerated concrete. In addition, the table includes the percentage of water absorption, expressed with respect to the material volume, measured at different testing times (10, 30, and 90 min), to provide a more comprehensive evaluation of the absorption behavior [12,61,62,63].

The experimental results highlight significant differences in the capillary water absorption behavior of the investigated materials, both in terms of absorption kinetics and capillary coefficient (*Cw,s*). More in detail, BIOAERMAC exhibits a markedly lower water uptake at all exposure times compared to BAAC and Commercial AAC, indicating a substantially reduced capillary absorption capacity.

Conversely, BAAC and commercial AAC show comparable trends, characterized by a rapid initial uptake followed by a progressive increase over time. This behavior is typical of highly porous cement-based materials with a well-developed and interconnected capillary pore network, in which water transport is governed by capillary suction and pore continuity [64].

The calculated *Cw,s* values further confirm this trend, with BIOAERMAC exhibiting a coefficient approximately three times lower than the other materials, while BAAC and commercial AAC display similar capillary performance. This result suggests that the reduced absorption of BIOAERMAC may be attributed to a refinement and/or partial discontinuity of the pore network, which limits water ingress and slows down capillary transport [65,66].

From a practical perspective, the lower capillary absorption of BIOAERMAC indicates an improved resistance to moisture penetration, which is commonly associated with enhanced durability performance in humid or aggressive environments, due to the reduced ingress of deleterious agents such as dissolved salts or pollutants [67].

### 3.3. Determination of Drying Shrinkage of Aerated Materials

In accordance with the standard, the conventional reference value of drying shrinkage (ε_cs,ref_) was determined from the regression curve as the difference in relative length variation between a moisture content by mass of μ_m_ = 30% and μ_m_ = 6%, and expressed in mm/m. The results are reported in Table 5.

**Figure 8:**
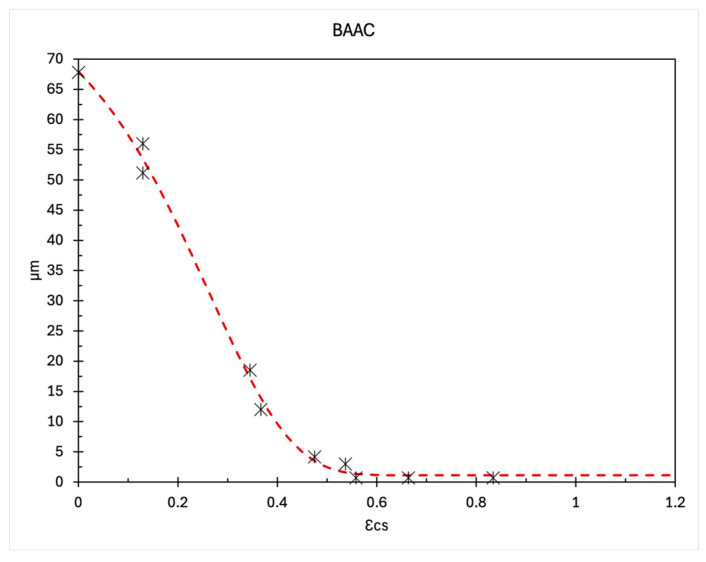
Relationship of shrinkage with moisture content expressed in mass % for BAAC. Experimental data are shown as cross markers, and the red dashed line represents the fitted trend.

**Figure 9:**
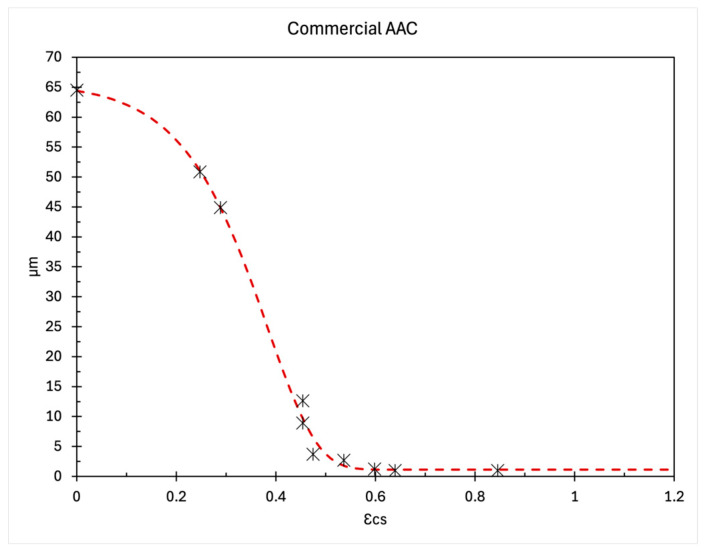
Relationship of shrinkage with moisture content expressed in mass % for commercial AAC. Experimental data are shown as cross markers, and the red dashed line represents the fitted trend.

**Figure 10:**
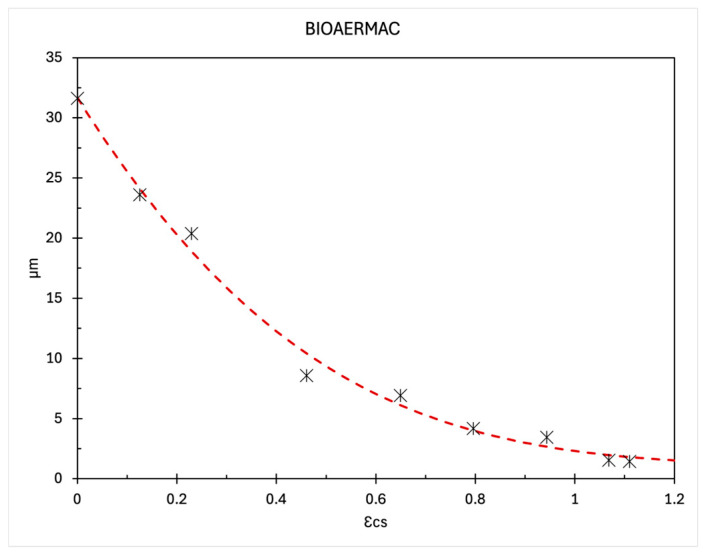
Relationship of shrinkage with moisture content expressed in mass % for BIOAERMAC. Experimental data are shown as cross markers, and the red dashed line represents the fitted trend.

Figure 8, Figure 9 and Figure 10 illustrate the evolution of shrinkage as a function of moisture content (evaluated by mass) for BAAC, BIOAERMAC, and commercial AAC, respectively. Each figure presents the experimental data points at which length measurements and specimen mass were recorded, along with the corresponding regression curves shown in red. This type of representation is in accordance with standard UNI EN 680:2006; a greater number of measurements were performed to enhance the graphical representation and obtain more representative data.

BIOAERMAC exhibits significantly higher drying shrinkage compared to the other two materials, with a value of 0.63 mm/m. The drying shrinkage values of BAAC and commercial AAC are similar.

**Figure 11:**
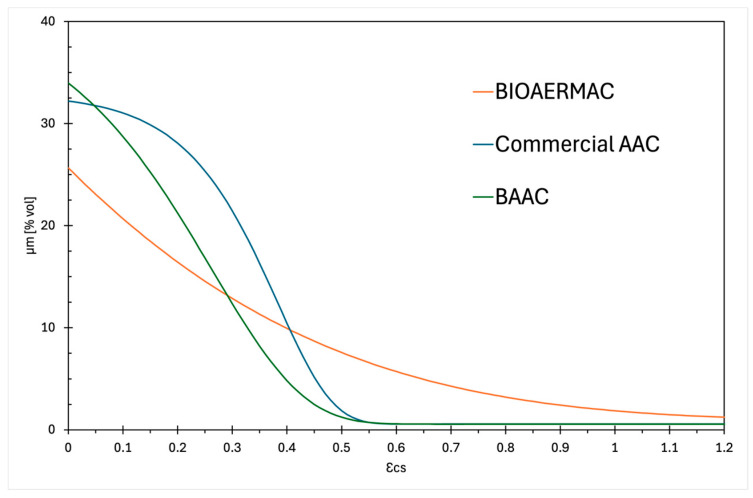
Relationship of shrinkage with moisture content expressed in volume % for different materials.

Figure 11 illustrates the variation of drying shrinkage as a function of moisture content for the three investigated materials. In this case, the water content is expressed as a percentage by volume (rather than by mass, as reported in Figure 8, Figure 9 and Figure 10) in order to account for the different densities of the materials and to allow for a more meaningful comparison. The graph indicates that the initial water content (after storage in water for 72 h) is higher for commercial AAC and BAAC specimens, with values of 32% and 33.5%, respectively, whereas BIOAERMAC exhibits a lower initial moisture content of 25.5%.

The increased water absorption observed in commercial AAC and BAAC is associated with the presence of a higher volume of voids (resulting from their lower density). Furthermore, the faster drying rate of these two materials compared to BIOAERMAC is mainly attributed to a greater degree of interconnection among the pores [49].

In cases where pores are less interconnected, as observed in the BIOAERMAC sample [31], air remains entrapped within the voids and does not contribute to water absorption, resulting instead in a slower drying process. The high porosity, often associated with an increased specific pore surface area, makes AAC specimens highly susceptible to shrinkage [10,68,69].

BIOAERMAC exhibits higher shrinkage but a lower capacity for water absorption, both in terms of volume and mass of the specimens. Commercial AAC and BAAC show similar behavior; however, BAAC exhibits a faster moisture loss rate. This aspect suggests that the absence of lime in the mixture, associated with the adoption of a green aeration method, plays a key role. It was found that shrinkage is instead almost independent of the dosage of aerating agent, silica fume and superplasticizer [68]. The progression of shrinkage over time is also influenced by external factors, including specimen dimensions and the ambient relative humidity [70]. Specifically, drying shrinkage is strongly influenced by environmental and geometrical factors, increasing under lower relative humidity conditions and being affected by the size and shape of the structural element [71].

## 4. Discussion

The obtained results consistently highlight the key role of pore structure in governing the hygrothermal and dimensional response of the investigated materials. Despite similar mechanical strength, the three materials exhibit different responses in terms of moisture transport and shrinkage, confirming that performance is primarily led by porosity characteristics rather than strength alone. It should also be noted that differences in density may contribute to overall behavior. However, the consistent correlation between moisture transport, shrinkage, and pore structure suggests that density alone cannot explain the experimental trends, highlighting the dominant role of pore connectivity and pore size distribution.

BAAC and commercial AAC show comparable performance in both hygroscopic and capillary absorption tests, characterized by high moisture uptake, pronounced hysteresis, and relatively low drying shrinkage. This combination is indicative of a highly interconnected pore network, in which both vapor diffusion and liquid transport are facilitated by pore continuity. Such a response is consistent with previous studies on autoclaved aerated concrete [43,44,47], where transport properties are governed by the connectivity and accessibility of capillary pores.

Conversely, BIOAERMAC exhibits significantly lower water absorption, both in vapor and liquid phases, together with reduced capillary coefficients, indicating limited pore connectivity and a more discontinuous transport network. In addition, this reduction may also be partially attributed to a more regular pore size and shape distribution, which further limits moisture transport processes. Such a microstructure limits fluid penetration and reduces transport dynamics, generally resulting in improved durability through the reduced ingress of aggressive agents. However, this reduced connectivity is accompanied by higher drying shrinkage, suggesting that moisture is retained within finer or partially isolated pores, where capillary stresses develop more intensely. Similar trends have been reported for cellular and foamed materials with refined pore structures, in which shrinkage is strongly influenced by internal moisture distribution and pore size [72,73,74].

This observation further indicates that total porosity alone does not govern dimensional response. Instead, the finer and less interconnected pore system limits internal moisture redistribution, leading to the development of higher moisture gradients and, consequently, increased capillary stresses within the material.

It should be noted that the observed differences in shrinkage may also be partially influenced by the intrinsic properties of the gypsum–CSA matrix, which differs from Portland cement-based systems. However, the comparison at similar mechanical strength and the consistent correlation with pore structure suggest that microstructural features observed for BIOAERMAC, characterized by more spherical and less interconnected pores, play a crucial role in controlling the observed behavior. While such a pore system reduces overall moisture uptake, it also limits internal moisture redistribution during drying. Therefore, localized moisture gradients may develop within the material, leading to higher internal stress concentrations in the solid matrix.

This mechanism provides a plausible explanation for the increased shrinkage observed in BIOAERMAC compared to BAAC and conventional AAC, where a more interconnected pore structure facilitates internal moisture redistribution, reduces moisture gradients, and enables partial relaxation of internal stresses.

Overall, the results demonstrate that the bioaeration approach enables effective control of pore structure, leading to distinct performance profiles. BAAC maintains performance comparable to conventional AAC, while BIOAERMAC introduces a different balance between reduced permeability and increased dimensional sensitivity. This trade-off highlights the importance of optimizing pore connectivity in bioaerated materials to achieve an optimal compromise between durability and dimensional stability.

## 5. Conclusions

This study investigated the hygrothermal and dimensional response of two innovative bioaerated materials (BAAC and BIOAERMAC) in comparison with commercial AAC, focusing on the role of pore structure under equal mechanical strength conditions. The main results can be summarized as follows:

- BAAC exhibits hygrothermal and capillary mechanisms comparable to conventional AAC, confirming the effectiveness of bioaeration as a sustainable alternative to aluminum-based foaming;
- BIOAERMAC shows significantly reduced moisture uptake, both in hygroscopic and capillary conditions (*Cw,s* approximately three times lower), indicating a less connected pore network and improved resistance to water ingress;
- Despite the lower absorption, BIOAERMAC demonstrates substantially higher drying shrinkage (≃0.63 mm/m), highlighting a trade-off between reduced permeability and increased dimensional sensitivity;
- The experimental results confirm that, regarding materials with similar mechanical strength, pore structure—particularly pore size distribution and connectivity—represents the controlling parameter for moisture transport and durability-related properties.

Overall, these findings provide a first experimental assessment of the hygrothermal and shrinkage behavior of these innovative materials, highlighting the fundamental role of pore structure in governing moisture transport and dimensional stability.

Future research will focus on a more detailed and direct investigation of pore structure through advanced microstructural characterization techniques, such as mercury intrusion porosimetry (MIP), and X-ray microtomography. These approaches will enable a quantitative evaluation of pore size distribution, connectivity, and their relationship with moisture transport and dimensional stability.

In addition, further experimental campaigns will address durability aspects under realistic service conditions, including freeze–thaw resistance, wet–dry cycling, resistance to aggressive environmental conditions and long-term hygrothermal aging, which are essential to validate the applicability of these materials in high energy efficiency buildings.

At the same time, recent literature highlights the growing interest in alternative aeration strategies for bio-based cellular concrete or low-density aerated materials [75,76]. These trends reflect the increasing focus on sustainable production processes and on the feasibility of non-conventional aeration methods. In this context, bioaeration approaches such as BAAC and BIOAERMAC represent a promising advancement toward safer and more sustainable cellular materials, in line with current research trends.

Future works will therefore aim not only to optimize the design of BAAC and BIOAERMAC but also to investigate these materials within a broader sustainability perspective, including environmental impact assessment through life cycle analysis (LCA), in comparison with conventional cellular concretes.

## 6. Patents

De Fazio, P.; Leter, G.; Lista, G. F.; Sposato, C.; Alba, M.B. Patent process for preparing bioaerated autoclaved cement, 2019, Patent WO/2019/049005.

De Fazio, P.; Sposato, C.; Alba, M. B.; Leter, G.; Feo, A. Process for preparing bioaerated composite materials, 2023, Patent WO/2023/152629 A1.

## Figures and Tables

**Table 1:** Comparative overview of BAAC and BIOAERMAC materials.

Parameter	BAAC (Bio Aerated Autoclaved Concrete)	BIOAERMAC (Bio-Aerated Gypsum-Based Composite)
matrix/binder system	Portland cement-based system, hardened under autoclave curing (AAC-type matrix)	gypsum–CSA based system (synthetic anhydrite + calcium sulfoaluminate cement), non-autoclaved
aeration mechanism	bioaeration via oxygen release from H_2_O_2_ catalyzed by *Saccharomyces cerevisiae*	bioaeration via oxygen release from H_2_O_2_ catalyzed by *Saccharomyces cerevisiae*
aerating agent	fully replaces aluminum powder traditionally used in AAC	no metallic aluminum powder required
curing process	autoclaving (≈180 °C, saturated steam pressure)	ambient/controlled curing (no autoclave)
porosity generation	in situ biogenerated closed and semi-closed pores, stabilized during autoclaving	in situ biogenerated pores, stabilized during hydration/setting of gypsum–CSA matrix
density range [kg/m^3^]	≃350–850	≃600–950
compressive strength [MPa]	comparable to commercial AAC at equivalent density classes (typically 2–6 MPa)	Up to ≃6 MPa depending on formulation and density
thermal conductivity [W/mK]	decreasing with density; values consistent with AAC insulation classes	≃0.15–0.30, with improvement at constant density when ELT is added

**Table 2:** Measured values of sorption curve established at a series of increasing equilibrium relative humidities at a given temperature in the range of 30% to 95% relative humidity.

Material Type	Sorption Curve—Moisture Content Volume by Volume *Ψ* [m^3^/m^3^]				
	30	45	60	75	95
BAAC	0.003415 ± 4.6 ×10^−5^	0.004888 ± 8.2 ×10^−5^	0.006526 ± 1.6 × 10^−4^	0.01371 ± 4.0 × 10^−4^	0.1130 ± 3.3 × 10^−3^
Commercial AAC	0.002839 ± 7.2 ×10^−5^	0.004004 ± 8.2 ×10^−5^	0.005069 ± 1.4 × 10^−4^	0.009009 ± 1.3 × 10^−4^	0.1310 ± 3.7 × 10^−3^
BIOAERMAC	0.003633 ± 1.0 × 10^−4^	0.005286 ± 8.2 ×10^−5^	0.007638 ± 1.5 × 10^−4^	0.01011 ± 1.3 × 10^−4^	0.07173 ± 2.5 × 10^−3^

**Table 3:** Measured values of desorption curve established at a series of increasing equilibrium relative humidities at a given temperature in the range of 30% to 95% relative humidity.

Material Type	Desorption Curve—Moisture Content Volume by Volume *Ψ* [m^3^/m^3^]				
	95	75	60	45	30
BAAC	0.1130 ± 3.3 × 10^−3^	0.02685 ± 1.2 × 10^−3^	0.01739 ± 6.0 × 10^−4^	0.01523 ± 4.5 × 10^−4^	0.01345 ± 4.2 × 10^−4^
Commercial AAC	0.1310 ± 3.7 × 10^−3^	0.018289 ± 8.0 × 10^−4^	0.01160 ± 4.4 × 10^−4^	0.01002 ± 5.3 × 10^−4^	0.01275 ± 7.7 × 10^−3^
BIOAERMAC	0.07173 ± 2.5 × 10^−3^	0.02791 ± 8.5 × 10^−4^	0.01579 ± 2.8 × 10^−3^	0.01250 ± 1.0 × 10^−3^	0.008872 ± 1.1 × 10^−3^

**Table 4:** Capillary absorption parameters of the investigated materials: *Cw,s* and volumetric water absorption (%) at different times (10, 30 and 90 min).

Material Type	*Cw,s*	Water Absorption [%] After		
	[g/(m^2^ × s^0.5^)]	10 min	30 min	90 min
BAAC	143 ± 13.8	6.0	8.2	11.3
Commercial AAC	157 ± 4.8	6.2	8.3	10.9
BIOAERMAC	50 ± 5.0	1.5	2.1	3.2

**Table 5:** Reference values of shrinkage calculated according to UNI EN 680:2006 standard.

	BAAC	Commercial AAC	BIOAERMAC
ɛ_cs,ref_ [mm/m]	0.16	0.12	0.63

## Data Availability

The original contributions presented in this study are included in the article/Supplementary Materials. Further inquiries can be directed to the corresponding author.

## Supplementary Materials

The following Supporting Information can be downloaded at: https://www.mdpi.com/article/10.3390/ma19132722/s1, Table S1: Measured values of sorption curve established at a series of increasing equilibrium relative humidities at a given temperature in the range of 30% to 95% relative humidity; Table S2: Measured values of desorption curve established at a series of increasing equilibrium relative humidities at a given temperature in the range of 30% to 95% relative humidity; Figure S1: Equilibrium moisture content curve mass by mass for BAAC; Figure S2: Equilibrium moisture content curve mass by mass for commercial AAC; Figure S3: Equilibrium moisture content curve mass by mass for BIOAERMAC.
