# Supplementary material for: Bioaerated Low-Density Composites from Industrial Byproducts: Advancing Carbon-Neutral and Energy-Efficient Material Systems in the Building Sector
Source: Materials (Basel). 2026 Jun 25;19(13):2722. doi: 10.3390/ma19132722 (PMC13363694; doi:10.3390/ma19132722)
Supplement: Supplementary file 1 [file materials-19-02722-s001.zip › materials-4374376-supplementary.pdf]

Article title:

Bioaerated Low-Density Composites from Industrial Byproducts: Advancing Carbon-Neutral and Energy-Efficient Material Systems in the Building Sector

Supplementary Materials

Table S1. Measured values of sorption curve established at a series of increasing equilibrium relative humidities at a given temperature in the range of 30% to 95% relative humidity.

Material type	Sorption curve - Moisture content mass by mass *u* [kg/kg]				
	30	45	60	75	95
BAAC	0.006177±1.1×10⁻⁴	0.008841±8.8×10⁻⁵	0.011803±5.6×10⁻⁵	0.024796±1.9×10⁻⁴	0.204432±3.3×10⁻³
Commercial AAC	0.005560±1.5×10⁻⁴	0.007840±1.6×10⁻⁴	0.009926±2.5×10⁻⁴	0.017643±2.7×10⁻⁴	0.256581±8.1×10⁻³
BIOAERMAC	0.004235±1.3×10⁻⁴	0.006162±1.1×10⁻⁴	0.008903±1.7×10⁻⁴	0.011790±1.5×10⁻³	0.083627±3.1×10⁻³

Table S2. Measured values of desorption curve established at a series of increasing equilibrium relative humidities at a given temperature in the range of 30% to 95% relative humidity.

Material type	Desorption curve - Moisture content mass by mass *u* [kg/kg]				
	95	75	60	45	30
BAAC	0.204432±3.3×10⁻³	0.048549±1.4×10⁻³	0.031440±3.4×10⁻⁴	0.027544±1.8×10⁻⁴	0.024331±1.8×10⁻⁴
Commercial AAC	0.256581±8.1×10⁻³	0.035813±1.6×10⁻³	0.022717±8.9×10⁻⁴	0.019632±1.1×10⁻³	0.024948±1.5×10⁻²
BIOAERMAC	0.083627±3.1×10⁻³	0.032538±9.5×10⁻⁴	0.018406±3.3×10⁻³	0.014570±1.2×10⁻³	0.010342±1.2×10⁻³

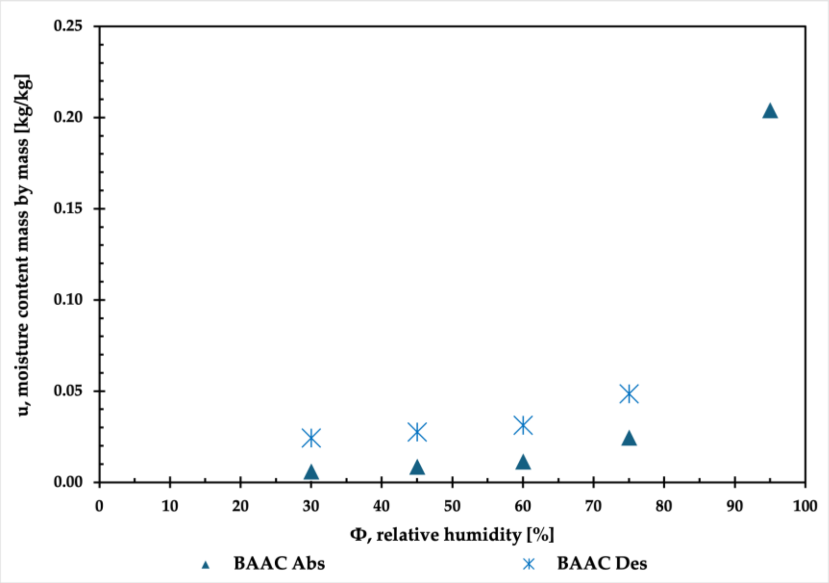

Figure S1. Equilibrium moisture content curve mass by mass for BAAC.

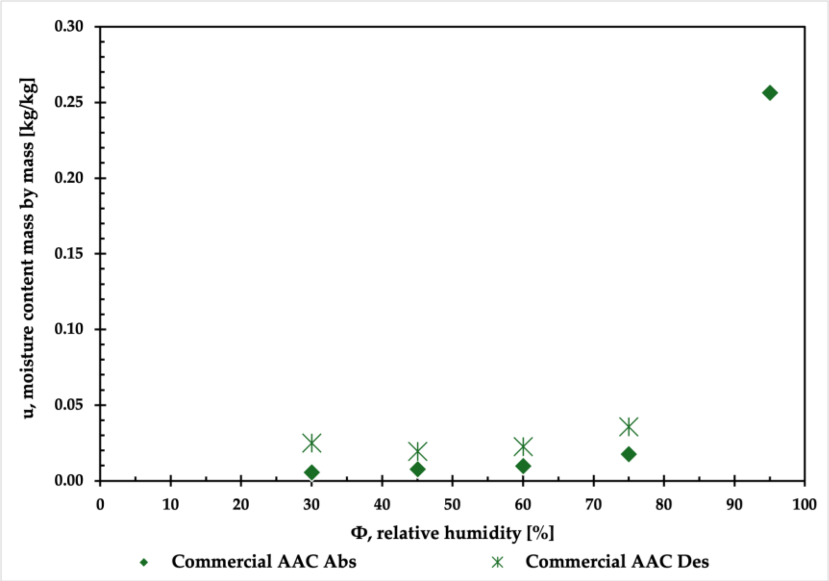

Figure S2. Equilibrium moisture content curve mass by mass for commercial AAC.

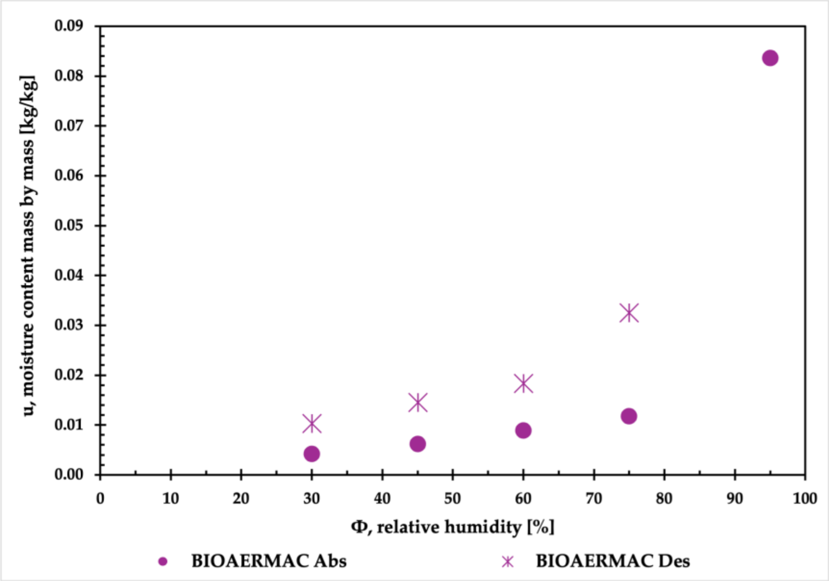

Figure S3. Equilibrium moisture content curve mass by mass for BIOAERMAC.
